# Noninvasive Characterisation of Foot Reflexology Areas by Swept Source-Optical Coherence Tomography in Patients with Low Back Pain

**DOI:** 10.1155/2013/983769

**Published:** 2013-03-11

**Authors:** Krishna Dalal, D. Elanchezhiyan, Raunak Das, Devjyoti Dalal, Ravindra Mohan Pandey, Subhamoy Chatterjee, Ashish Datt Upadhyay, V. Bharathi Maran, Jyotirmoy Chatterjee

**Affiliations:** ^1^Department of Biophysics, All India Institute of Medical Sciences, Ansari Nagar, New Delhi 110 029, India; ^2^School of Medical Science and Technology, Indian Institute of Technology, Kharagpur, West Bengal 721 302, India; ^3^Department of Biostatistics, All India Institute of Medical Sciences, Ansari Nagar, New Delhi 110 029, India; ^4^Indian Institute of Astrophysics, Bangalore, India

## Abstract

*Objective*. When exploring the scientific basis of reflexology techniques, elucidation of the surface and subsurface features of reflexology areas (RAs) is crucial. In this study, the subcutaneous features of RAs related to the lumbar vertebrae were evaluated by swept source-optical coherence tomography (SS-OCT) in subjects with and without low back pain (LBP). *Methods*. Volunteers without LBP (*n* = 6 (male : female = 1 : 1)) and subjects with LBP (*n* = 15 (male : female = 2 : 3)) were clinically examined in terms of skin colour (visual perception), localised tenderness (visual analogue scale) and structural as well as optical attributes as per SS-OCT. From each subject, 6 optical tomograms were recorded from equidistant transverse planes along the longitudinal axis of the RAs, and from each tomogram, 25 different spatial locations were considered for recording SS-OCT image attributes. The images were analysed with respect to the optical intensity distributions and thicknesses of different skin layers by using AxioVision Rel. 4.8.2 software. The SS-OCT images could be categorised into 4 pathological grades (i.e., 0, 1, 2, and 3) according to distinctness in the visible skin layers. *Results*. Three specific grades for abnormalities in SS-OCT images were identified considering gradual loss of distinctness and increase in luminosity of skin layers. Almost 90.05% subjects were of mixed type having predominance in certain grades. *Conclusion*. The skin SS-OCT system demonstrated a definite association of the surface features of healthy/unhealthy RAs with cutaneous features and the clinical status of the lumbar vertebrae.

## 1. Introduction

In the context of establishing the scientific basis of noninvasive therapeutic technique such as reflexology, it is important to elucidate the structural and functional features of the reflexology areas (RAs). It is also important in improving the diagnostic and prognostic values of the mentioned healing methodology in different clinical conditions [1–3]. In reflexology, it is assumed that the particular body surface areas (which are interconnected to the internal body parts and maneuverable) manifest the functional status of the target organs and accordingly carry the characteristic physical signatures. The externally recordable surface features, which are observed either in single or in combination on the RAs, are stated as follows: (i) tenderness in response to pressure [2, 3], (ii) the skin colour (reddish brown, brown/dark brown, black), (iii) the texture (namely, scaling, cracking skin, recurring corns) [4], (iv) concavity, convexity formation (puffiness or swelling, depression) [5], (v) a rise in the localised temperature [6, 7], (vi) a change in electrical impedance [8], and (vii) the perceived presence of tiny granules [9, 10]. The methods for clinically assessing these RA changes suffer from subjectivity and thus pose certain limitations and thereby may mislead the reflexology researchers/practitioners when making conclusions about their observations [4, 11]. With this in mind, this study was conducted to explore the noninvasive characterisation of the RAs mapped on the feet as related to the lumbar vertebrae (as per existing literatures [6, 12]) by skin swept source-optical coherence tomography (SS-OCT) [13–15] in normal subjects and patients suffering from low back pain (LBP) [16]. 

Epidemiological studies have revealed that among musculoskeletal disorders, LBP is a common symptom and many subjects have reported experiencing LBP at least once in their life [17]. A study examining 11234 patients with LBP indicated that the occurrence of LBP in working adults of rural North India was approximately 23.09% [18]. The incidence of back pain amongst the long-distance truck drivers of the mountainous areas of Sikkim and in Indian nursing students was reported to be 73.52% [19] and 58.7% [20] respectively. From the existing literature, it is also obvious that nonspecific LBP has become a major modern health problem worldwide. It has been reported that the lifetime prevalence of LBP is approximately 84%, and the prevalence of chronic LBP is nearing to 23%, with 11-12% of the population disabled by the condition [21]. Most cases of LBP do not require urgent care, but pain that radiates or is referred to the other lower limb may be excruciating enough to necessitate emergency medical care. The symptoms and severity of LBP may vary greatly and many times are ignored at the onset of the disease. However, the early diagnosis and noninvasive management of pain are of primary importance in getting relief as well as in efficiently treating patients. 

In this context, skin SS-OCT, a real-time imaging technique with micron-scale resolution and a few millimeter of field of view, offers great promise for the noninvasive characterisation of integument in health and disease with the ability to more deeply and swiftly elucidate epidermal and dermal structures [22, 23]. Thus the technique is especially applicable to the characterisation of RAs. In SS-OCT, a tunable wavelength of laser source, whose wavelength rapidly sweeps within a selected wavelength band, allows the spectrum of the interferometer output to be recorded sequentially through a single detector. In assessing SS-OCT image features, based on relative optical signal intensity, the term lucidity (brightness/luminosity) is used. Less hydrated tissues reflect more light due to back scattering and become more lucid in contrast to the low-lucidity features of areas with higher fluid or fat content [24]. Furthermore, stratified skin layers demonstrate varied optical properties according to their refractive indices, relative to the thickness and organic/inorganic compositions of the anatomical sites and regeneration phases [25].

With this in mind, this study applied SS-OCT imaging to elucidate the surface and subsurface features of RAs in patients with LBP and in normal healthy subjects (without LBP) as well as to draw correlation with the related clinical observations. 

## 2. Materials and Methods

### 2.1. Location of the Study and Settings

The study was conducted among the Indian population at a tertiary care medical institute of New Delhi and at the Indian Institute of Technology, Kharagpur, India. The data were collected under the guidance of an experienced reflexology scientist and with the informed consent of the subjects. The SS-OCT data were captured and interpreted by the domain knowledge experts. All the possible biases involved in the data collection and analyses were minimised by utilising the different expertise of the various authors who worked independently and were blinded to the relationship between the data recordings and clinical findings.

### 2.2. Selection of the Subjects

The subjects were engaged in academic activities. Health scientists and professionals with and without a history of nonspecific LBP were selected to take part in this study upon explaining the implications of this noninvasive study and under ethical clearance. Subjects of both genders, aged 24 years to 45 years and with 18.5 < Body Mass Index (BMI) <29, participated in this study. This study included a control sample size of 6 healthy volunteers (male and female ratio = 1 : 1) and a sample size of 15 subjects (male : female = 2 : 3) suffering from LBP. The patients reported to be suffering from LBP due to the prolonged duration of incorrect seating postures, herniated discs and spinal stenosis. None of the study subjects were suffering from fractures, scoliosisor kyphosis, osteoporosis, osteoarthritis, or bacterial infection. The pain scores involved in the LBP were recorded by research scientists using a visual analogue scale (VAS) score with a scale of “0” to “10” (with “0” being “no pain” and “10” being “unbearable pain”). There was no participant who reported “unbearable pain” in the study. The reflexology areas of the subjects without any history of LBP were considered to be “normal” (controls). 

### 2.3. Study Protocol Requirements for Subjects' Preparation prior to Data Collection

 The importance of subjects' participations in the study and the need for maintaining a particular study protocol were explained to the subjects. They followed the standard preexamination preparatory protocol for avoiding dehydration, maintaining uniform stable blood circulation, and keeping feet optically clean. After arrival at the laboratory, they were asked to remove the footwear's and to sit comfortably and relaxed in the long sitting position with back support [26] for 15 minutes so as to achieve uniform blood circulation throughout the body. At the end of this stipulated time, they were advised to drink 250 mL of water and to wipe off their feet using a clean and wet (with water) cloth to remove the dart particles and oily substances. This method facilitated the reflexology scientists to find optically clear skin. The feet were left to be air dried and untouched for another 15 minutes to establish uniform micro-circulation of blood. The optically clear skin and the absence of oily substances on the examined skin areas were reconfirmed by physical observations too. This procedure was conducted at a room temperature of 25°C. 

### 2.4. Physical Assessment of the RAs

A foot reflexology map for the lumbar vertebrae was used following the existing literatures [27] and using the maps from the Indian School of Reflexology Practice [12] together with the in-house data records generated through various research projects that had noted the abnormal signatures [6]. The physical features on the surface skin of lumbar RAs were examined clinically and independently by a research scientist under the guidance of the methods of “Eunice D. Ingham Stopfel” [28], Kevin and Barbara Kunz [29], and an in-house reflexology program. The following observations were made: (i) tenderness under a defined finger pressure as assessed by a pedography system (Emed-AT/2, Novel GmbH, Germany [30]) in the range from 30 N /cm^2^ to 35 N/cm^2^ and (ii) the skin colour changes. A VAS score with the scale of “0” to “10” was used to determine the localised pain of the RAs in response to defined finger pressure on the RAs.

### 2.5. Optical Image Data Recordings

 The images of the skin, with original skin colour (normal) and with change in skin colour (abnormal), were recorded using an optical camera (Nikon D200, Japan). Prior to capturing the photographs, the subjects were rechecked to ensure their feet optically clear, particularly of dirt and oily substances, to avoid optical disturbances of the images due to the rays reflected from well-defined areas. 

### 2.6. SS-OCT Instrumentation

Instrumentation SS-OCT (OCM1300SS, Thorlabs Incorporated, NJ, USA) consists of a high-speed frequency-swept external cavity laser. The central wavelength of the laser source is 1325 nm, and it has half-power spectral bandwidth > 100 nm and an average output power of 10 mW. The axial scans (A-scans) of laser were performed at a sweeping frequency of 16 kHz to construct a depth profile of *∼*1.75 mm. The system has two interferometers, a Michelson interferometer and a Mach-Zehnder interferometer (MZI, Thorlabs INT-MZI-1300), as well as a broadband coupler (Thorlabs, FC1310-70-50-APC). The skin SS-OCT system produces cross-sectional images for different subsurface layers. The details of the instrumentation were previously described [31]. 

### 2.7. SS-OCT Image Capturing and Data Analyses

**Figure 1:**
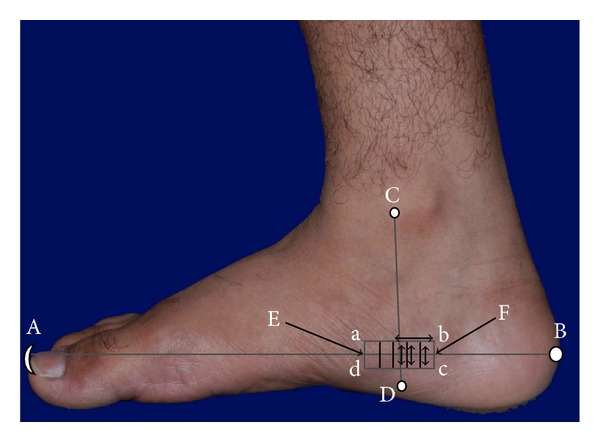
The medial view of lumbar vertebrae reflexology area “abcd” where ab = cd = 3 cm; ad = cb = 1 cm. Each of A, B, C, and D is the reference point for this reflexology area, where the line CD is perpendicular to “AB”. A: tip of big toe; B: midpoint of posterior surface of calcaneum; C: anterior border of medial malleolus; D: 1.5 cm below Sustentaculum tali. E is the perpendicular bisector of “ad” and F is that of “bc”.

The sub-cutaneous structures of normal and abnormal RAs were recorded using the SS-OCT imaging system as described. The IIT Kharagpur researchers were involved in SS-OCT imaging within their respective domains of knowledge [31]. Just before capturing data, a drop of glycerol was applied with a very light touch uniformly over the skin to minimise optical mismatch without affecting stratum corneum integrity [32]. Each reflexology area for the lumbar vertebrae (the rectangular area marked “abcd” in Figure 1) was divided into 5 equal subareas as shown in Figure 1. For each foot, 3 image frames were collected along the axis vertical to the longitudinal axis (EF) from 3 segmental areas, as shown by the two-way arrows (Figure 1), and the scanning length was 10 mm. All the LBP subjects exhibited abnormality within this zone of the referred RA. Hence from each subject, 6 optical tomograms were recorded and each tomogram contributed 25 data records. The handheld probe of the SS-OCT system was held vertically over the specified areas during imaging. The grey-scale 2D images were recorded at a resolution of 512 × 512 pixels with a pixel resolution of 8.42 **μ**m/pixel and stored for analyses. The pixel resolution was calculated using the data supplied by the manufacturer (i.e., 12 **μ**m is the axial resolution in air and 1.4 is the refractive index of skin) [33]. The optical intensity distributions and thicknesses of different skin layers were determined independently by using AxioVision Rel. 4.8.2 software (Carl Zeiss, Germany, 2010).

The LBP grades were categorised depending on the subjective pain intensity using VAS scores and the duration of disease (LBP).

**Figure 2:**
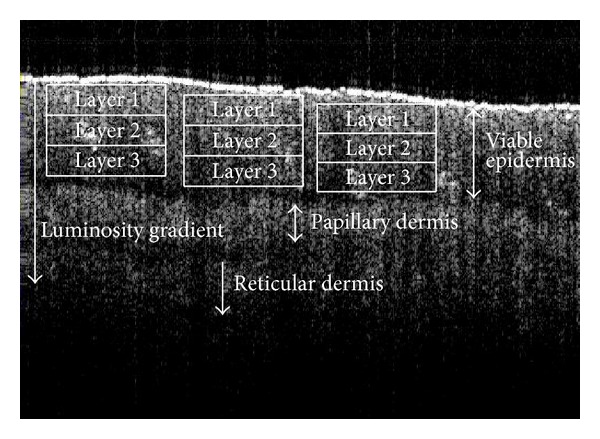
A scheme of data collection on the luminosity of the different layers of the cutaneous skin related to the reflexology area of lumbar vertebrae.

To produce a grey-scale intensity gradient of a particular SS-OCT image along the depth of the viable epidermis (i.e., from the layer adjacent to stratum corneum towards the basal membrane), each epidermal depth was divided into 3 equal layers as demonstrated in Figure 2. The average values of the pixel luminosities of each layer of a viable epidermis, papillary dermis and reticular dermis, were measured by employing AxioVision 4.8.2 software. The thickness of the stratum corneum (SC) and the depth of the viable epidermis (VEP) were measured using the same software, and these were recorded in terms of pixels. The pixel depths were then converted into the dimension of length (micrometer (*μ*m)) using the pixel resolution (i.e., 8.42 **μ**m/pixel). The SS-OCT images were categorised into 4 grades depending on the characteristics of the pathological conditions of the same. Based on the previous domain knowledge in interpreting skin SS-OCT images and skin histology, this grading was proposed by the related experts [31]. The grading was mainly based on the optical intensity parameters of the individual skin layers and the extent of the distinctly separable layers of the targeted skin areas. Changes in the optical intensity parameters were noted through the comparison of different SS-OCT images. The images exhibiting all the distinctly visible layers were defined as normal and described as grade 0. The extent of deviation from this category in terms of distinction in the visibilities of layers and magnitude of lucidity led to the designation of RA abnormality grades, such as, grade 1, grade 2 or grade 3 of abnormality.

### 2.8. Statistical Data Analyses

**Figure 3:**
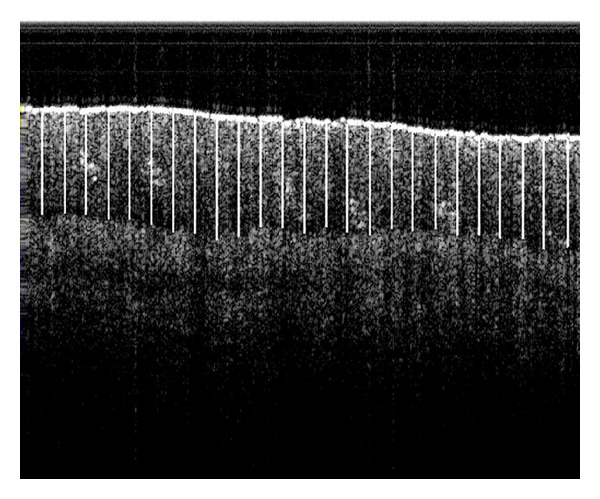
A demonstration of the method for collecting image data records. The SS-OCT image frame consists of 25 equidistant vertical line segments within the viable epidermis. Each line represents the depth of the viable epidermis (in pixels) at a particular location.

With respect to the sample size of the images considered for the analysis, it may be noted that 126 SS-OCT image frames were captured from 21 subjects. And from each image frame, the depth of the VEP was measured from 25 equidistant spaces along the horizontal axis (an example is shown in Figure 3.). In other words, a total of 3150 data records was analysed in estimating the depth of the viable epidermis and another set of 3150 data records was utilised to determine the thicknesses of the stratum corneum which were also measured from the identical locations. It may also be recalled that the subjects included in the study were declared to be healthy (normal subjects) or unhealthy (LBP patients). But it was interesting to note that according to the proposed RA grades (i.e., 0, 1, 2, and 3) most of the study subjects (90.5%) could not exhibit absolutely normal (i.e., grade 0) or absolutely diseased (i.e., within grades 1–3) RAs. Instead, the features indicated mixed characteristics, with the predominance of certain grades. Therefore, in the analyses, every image frame was treated as a sample, and statistical analyses were accordingly performed.

The data associated with individual SS-OCT image followed the normal distribution and hence the mean of each image frame data record was used for analyses. The mean values of the data records for estimating either the thickness of the SC or the depth of the VEP did not follow the normal distribution, and hence, the median (range) of each variable was used for analyses. A two-sample Wilcoxon rank-sum (Mann-Whitney) test was used to compare the median values between males and females for the different grades. Kruskal-Wallis equality-of-populations rank test was used to compare the median values of the depth of the viable epidermis and that of the thickness of SC according to grades for males and females separately. STATA 11.2 (special edition) statistical data analysis (StatCorp, TX, USA) software was used for the data analyses. In this study, *P* values less than 0.05 were considered as statistically significant. 

## 3. Results 

**Figure 4:**
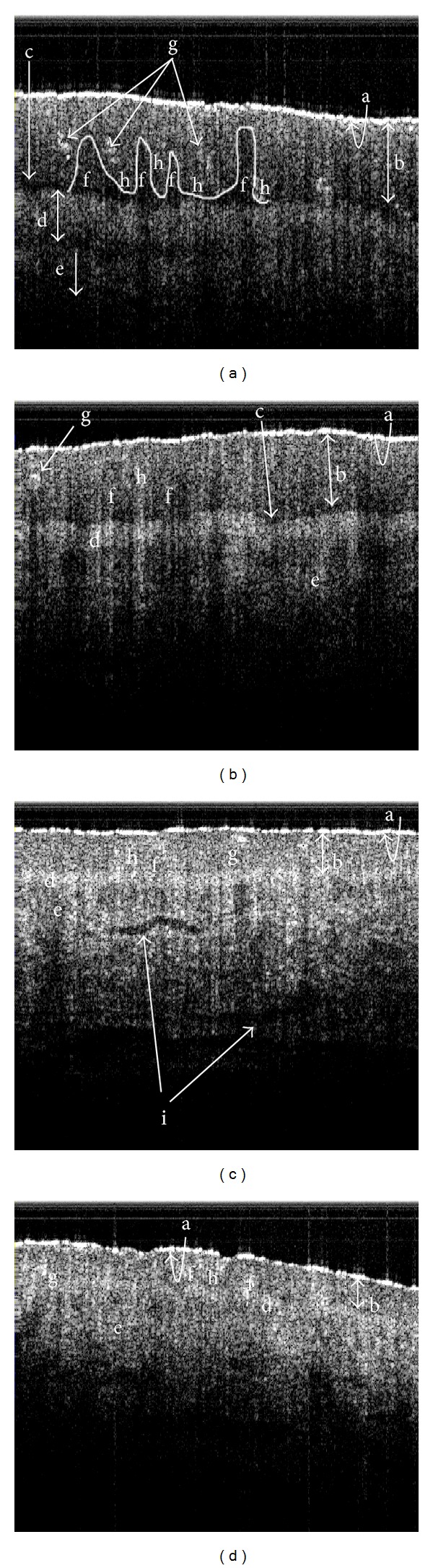
The skin SS-OCT images of lumbar reflexology areas under different pathological conditions: (a) grade 0 (normal); (b) grade 1 (early degeneration); (c) grade 2 (moderate degeneration); (d) grade 3 (severe degeneration). a: stratum corneum; b: viable epidermis; c: basal membrane; d: papillary dermis; e: reticular dermis; f: dermal papillae; g: rete pegs; h: sweat glands; i: macrovessels.

**Figure 5:**
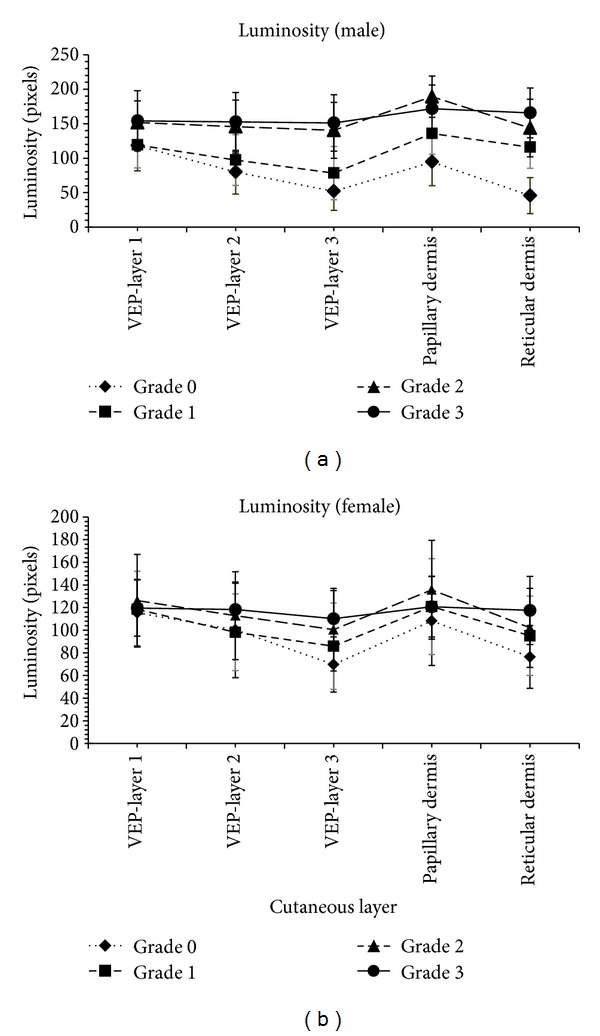
The graphical variations of the luminosity of cutaneous layers under the different grades of the degenerations of the RAs.

The demographic data together with the representatives of different grades of SS-OCT images are shown in Table 1. The skin SS-OCT images in the lumbar RAs could be clearly delineated into four optically distinct regions and their intersections: stratum corneum (SC), viable epidermis (VEP), papillary dermis (PD), and reticular dermis (RD). Other features such as, basal membranes (BMs), rete pegs, dermal papillae, macrovessels, and sweat glands were also visible (Figures 4(a)–4(d)). However lucidity was altered in these features, which could be corroborated with plausible pathological changes. The variations of lucidity in the different cutaneous layers of both genders are shown in Figure 5. The range of luminosity observed in males was from 26 pixels to 199 pixels and that in case of females was found to be from 23 pixels to 156 pixels. The SC region and the top layer of the VEP adjacent to the SC in normal RAs were hyper-lucid, with a smooth surface (Table 1, Figures 4(a) and 5). In subjects with LBP, optical distinctness, in terms of lucidity among different layers, was reduced and luminosity increased with the higher grades of abnormalities (Figure 5). In such cases, the intensity of the papillary dermis was remarkably increased in all abnormal RA grades as compared with that of normal (grade 0) RAs and the changes are explicitly indicated in Figures 4(b)–4(d) and Figure 5 respectively. Furthermore, based on the depth of the VEP and the thickness of the SC, gender could be distinguished, as elaborated in Tables 2 and 3 respectively.

Given the fine differences in the SS-OCT features of the epithelial and subepithelial regions, the tomograms of the RAs of LBP patients could predominantly belong to any one of the 3 abnormal categories as explained in the sub-section *“SS-OCT image capturing and data analyses*”. In grade 1 RAs, the distinction between the papillary and reticular dermis started to be less distinct, giving rise to mild changes in images (Figures 4(b) and 5 and Table 1). Such subjects had a predominantly a clinical history of mild LBP (0.5 < VAS ≤ 3) from long (duration > 3 hours) seating/standing. In grade 2 RA subjects, who had a clinical history of occasional moderate pain (3 < VAS ≤ 6) of 6 months < disease duration < 3 years, lucidity increased in the dermoepidermal junction, and the papillary dermis was not delineable from the reticular dermis; the SC roughness was also increased (Figures 4(c) and 5 and Table 1). Such cases were designated as having moderate changes in the SS-OCT images. In grade 3 subjects, the roughness of the SC was increased, and the optical properties of the epithelial and subepithelial dermis became almost homogeneous (Table 1, Figures 4(d) and 5). Such cases were recorded as severe changes (grade 3) in the SS-OCT images. These cases were associated with a clinical history of chronic pain with frequent severity (6 < VAS ≤ 9) needing medical intervention. Tables 2 and 3 depict, respectively, the statistically significant deviations of the depths of the VEP and the thicknesses of the SC for abnormal reflexology areas and the normal reflexology areas within the individual genders. The results were highly significant statistically in all situations, except the case of the thickness of the SC when comparing grade 0 and grade 1 (Table 3). 

The present study did not explore the dependence of the observed physical features and the subsurface structures of the lumbar reflexology areas with BMI variation. 

## 4. Discussion

This study explored the efficacy of skin SS-OCT in illuminating the subsurface structures of the special cutaneous areas (marked as lumbar RAs) on feet. In particular, the capturing of micron level information for optical signatures was relevant to the related surface areas as well as the clinical findings. Therefore, this approach bears significance in integrating the understanding and validating the assumptions embedded in the concept of reflexology that the surface features have a crucial association with the status of subsurface features in normal or abnormal RAs. H. Marquardt [34] stated that while clinically evaluating the feet, using the method of reflexology, the practitioners should not overlook or forget the subject's reflexology areas that were abnormal upon visual examination. 

 In this study, the lumbar RAs of the patients suffering from the different grades of LBP were evaluated with reference to the identical parameters of the normal subjects. Here, the SS-OCT demonstrated 4 distinctly delineable zones in the vertically downward directions of normal skin and successfully distinguished gender-specific variations (Figures 4(a), 4(b) and 5, Tables 1, 2, and 3). This subsurface imaging tool efficacy has become useful in recording alterations to the structural organisations and the lucidities of the skin layers in the studied reflexology skin areas in patients with LBP. Interestingly, changes in the SC layer in terms of increased roughness were demonstrated by the SS-OCT images and these changes could be associated with a greater dehydrated status (Figures 4(a)–4(d) and Table 1). The elucidation of the luminosity changes in the VEP of LBP patients was also remarkable. In normal RA (grade 0), the VEP layer adjacent to the SC (the layer 1 of Figure 2) possessed maximum lucidity (Figures 4(a) and 5). In patients with mild LBP (0.5 < VAS score ≤ 3 with a duration of disease < 6 months), and with moderate LBP (3 < VAS score ≤ 6 with a duration of disease of 6 months to 3 years), the luminosity of the VEP increased relative to a normal RA, and maximum luminosity was observed in the papillary dermis (Figures 4(b)–4(c) and Figure 5), which was different from what was observed with grade 0 RAs (normal). A drastic change was noted in the case of the RAs (grade 3 abnormality) associated with severe LBP in which luminosity throughout the VEP, the papillary dermis and the rest of the dermis became almost uniform (Figures 4(d) and 5). This probably indicated some compositional impairment in this part of epidermis. Furthermore, in LBP patients who reported severe clinical symptoms, the distinction between the VEP and the subepithelial dermis of abnormal RAs started to become less clear. In the diseased conditions, the dermoepidermal junction became highly lucid in contrast to the normal junctions with low lucidity. This type of change in lucidity was definitely indicative of changes in the density of the papillary dermis. 

In addition, the SS-OCT features nicely demonstrated the gradual thinning down of the VEP layer based on the severities of the diseased conditions (mild, moderate and severe) in concurrence with the sub-epithelium changes (grade 1, grade 2 and grade 3 respectively for abnormal RAs as depicted in  Tables 2 and 3). Hence, this study was efficient in exploring the correlation between the clinical observations of the RAs in LBP patients and their subsurface features. It was noticed in patients with LBP, irrespective of the severity of pain and duration of disease, that there was a feeling of pain under finger pressure in abnormal RAs, and the intensity of tenderness increased with the severity of LBP. This observation could also be correlated with the epithelial and subepithelial changes observed in the SS-OCT images. The SS-OCT features depicting the increased luminosity of the papillary dermis could have also indicated increased stiffness and dehydration. Therefore, under pressure a localised pain sensation in abnormal RAs increased. This hypothesis concerning tenderness following the application of finger pressure to an abnormal reflexology area is also supported by the reports of the presenting authors [6] and other researchers working in the field of reflexology [34–36]. Furthermore, the signatures of the enhanced diameters of the blood vessels in the abnormal RAs recorded by the SS-OCT images may explain the observation of K. Kunz and B. Kunz [37] who mentioned that the pooling of lymph fluid below the skin is one of the internal forms of blockage in a reflexology zone. In this context, it is obvious that these accumulations may reflect darker skin colour, which may be visible externally as an abnormal feature. 

## 5. Conclusion

This clinical fundamental study on exploring the cutaneous features of RAs by noninvasive SS-OCT was successful in recording the microanatomical alterations of RAs in subjects suffering from LBP and also demonstrated the possibility of compositional variations that clarify structural changes as well as clinical findings. Furthermore, with an in-depth study, particularly one that uses polarisation-sensitive OCT, these compositional variations could be validated, and a more direct correlation between the surface and subsurface compositions could be established. Therefore, it may be concluded that SS-OCT offers a new challenge for reflexology researchers in validating the belief of reflexology experts that the surface features have a definite correlation to the structural and functional status of the related internal body parts. 

## Figures and Tables

**Table 1:** Optical and SS-OCT images of lumbar reflexology areas and corresponding pain history.

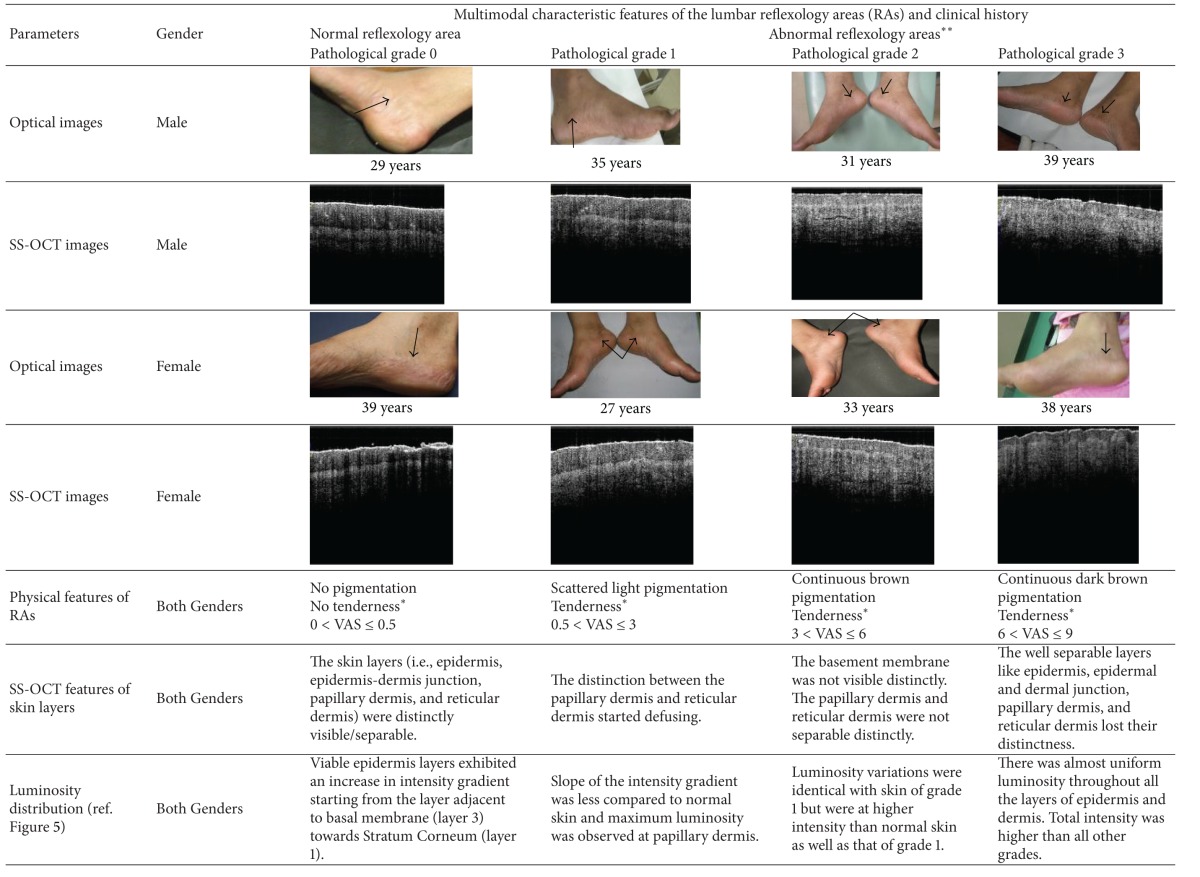 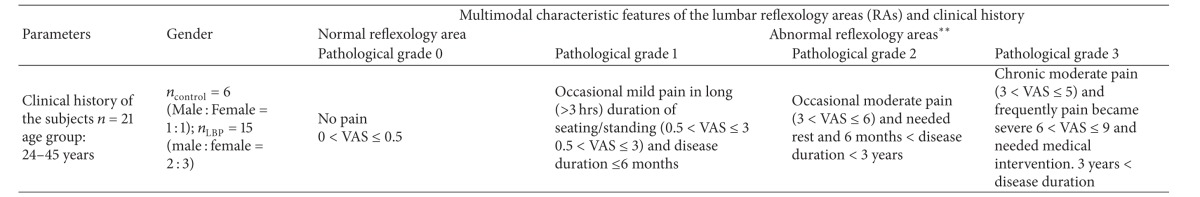

The depth of subcutaneous layer for which SS-OCT images were captured was 1.75 mm.

*Tenderness was observed in response to finger pressure (FP) (30 N/cm^2^ < FP < 35 N cm^2^).

**Tenderness without pigmentation *n*% = 41%; tenderness with pigmentation *n*% = 59%; pigmentation without tenderness *n*% = 0; *P* < 0.05.

SS-OCT feature-based pathological grades of lumbar reflexology areas: grade 0: normal RAs; grade 1 RAs: early degenerative changes in SS-OCT images; grade 2 RAs: moderately advanced changes in SS-OCT images; grade 3 RAs: advanced changes in SS-OCT images.

**Table 2:** Comparison of the depth of viable epidermis.

Grade	Gender				*P* value (in between male and female SS-OCT images)
	Male		Female	
	Sample size of image data (*n*)	Depth of viable epidermis (*μ*m) Median (range)^†^	Sample size of image data (*n*)	Depth of viable epidermis (*μ*m) Median (range)^†^
Grade 0	300	942 (481–1113)	325	575 (497–1170)	0.001
Grade 1	250	820 (354–990)	500	532 (380–676)	0.001
Grade 2	450	602 (287–790)	650	397 (258–569)	0.001
Grade 3	275	242 (134–465)	400	259 (148–468)	0.248
Overall *P* value		0.001		0.001	
Post hoc multiple grade comparison					
0 versus 1		0.001		0.001	
0 versus 2		0.001		0.001	
0 versus 3		0.001		0.001	
1 versus 2		0.001		0.001	
1 versus 3		0.001		0.001	
2 versus 3		0.001		0.001	

^†^Values are expressed as median (minimum–maximum).

**Table 3:** Comparison of the thickness of stratum corneum.

Grade	Gender				*P* value (in between male and female SS-OCT images)
	Male		Female	
	Sample size of image data (*n*)	Thickness of stratum corneum (*μ*m) Median (range)^†^	Sample size of image data (*n*)	Thickness of stratum corneum (*μ*m) Median (range)^†^
Grade 0	300	44.8 (37.9–53.9)	325	41.7 (31.6–51.0)	0.001
Grade 1	250	44.2 (28.7–54.7)	500	40.0 (34.1–49.2)	0.001
Grade 2	450	35.2 (23.6–45.9)	650	25.7 (17.3–39.6)	0.009
Grade 3	275	18.5 (10.5–22.3)	400	11.2 (6.0–17.7)	0.023
Overall *P* value		0.001		0.001	
Post hoc multiple grade comparison					
0 versus 1		0.363		0.679	
0 versus 2		0.003		0.001	
0 versus 3		0.001		0.001	
1 versus 2		0.109		0.001	
1 versus 3		0.004		0.001	
2 versus 3		0.008		0.001	

^†^Values are expressed as median (minimum–maximum).
